# Supplementary material for: Direct nucleic acid analysis of mosquitoes for high fidelity species identification and detection of Wolbachia using a cellphone
Source: PLoS Negl Trop Dis. 2018 Aug 30;12(8):e0006671. doi: 10.1371/journal.pntd.0006671 (PMC6116922; doi:10.1371/journal.pntd.0006671)
Supplement: S1 Table — (PDF) [file pntd.0006671.s001.pdf]

S1 Table. Primers, probes, and target sequences for *A. aegypti coi* and *Wolbachia w*AlbB/*w*Pip *wsp* LAMP-OSD assays.

	Name	Sequence
Wolbachia wsp LAMP-OSD assay	*Wolbachia w*AlbB Surface Protein (*wsp*) gene target	TGCCTATCACTCCATACGTTGGTGTTGGTGTTGGTGCAGCATATATCAGCAATCCTTCAGAAGCTAGTGCAGTTAAAGATCAAAAAGGATTTGGTTTTGCTTATCAAGCAAAAGCTGGTGTTAGTTATGATGTAACCCCAGAAATCAAGCTTTATGCTGGTGCTCGTTATTTTGGTTCTTATGGTGCTAGTTTTAATAAAGAAACAGTATCAGCTACTAAAG
	WSP.F3	TGCCTATCACTCCATACGT
	WSP.B3	CTTTAGTAGCTGATACTGTTTCT
	WSP.FIP	TGCTTGATAAGCAAAACCAAATCCTGGTGCAGCATATATCAGCAA
	WSP.BIP	AGCTGGTGTTAGTTATGATGTAACCCCACCATAAGAACCAAAATAACGAG
	WSP.LF	CCAGAAATCAAGCTTTATGCTGGTG
	WSP.OSD.FAM	TCCTTCAGAAGCTAGTGCAGTTAAAGATCAAAAAGCAC/36-FAM/
	WSP.OSD.Q	/5IABkFQ/GTGCTTTTTGATCTTTAACTGCACTAG/3InvdT/
Aedes aegypti coi LAMP-OSD assay	*Aedes aegypti* cytochrome oxidase I (*coi*) gene target	CCGGATTTGGAATAATTTCTCATATTATTACTCAAGAAAGTGGGAAAAAGGAAACATTTGGAACTTTAGGAATAATTTATGCTATATTAACAATTGGATTATTGGGATTTATTGTTTGAGCTCATCATATATTTACAGTAGGTATAGACGTAGATACTCGAGCTTATTTTACTTCAGCAACTATAATTATTGCTGTTCCTACAGGAATTAAAATTTTTAGTTGATTAGCAACTTTACACGGAACTCAA
	AE.COI.F3	CCGGATTTGGAATAATTTCTCA
	AE.COI.B3	TTGAGTTCCGTGTAAAGTTG
	AE.COI.FIP	TGATGAGCTCAAACAATAAATCCYACTCAAGAAAGYGGRAAAAAGG
	AE.COI.BIP	TAGGTATAGACGTAGATACTCGAGCATTCCTGTAGGAACAGCAATA
	AE.COI.LF	GCTTATTTTACTTCAGCAACTATAAT
	AE.COI.OSD.FAM	GAAACATTTGGAACTTTAGGAATAATTTATGCTATATTAACAATTGGAGC/36-FAM/
	AE.COI.OSD.Q	/5IABkFQ/GCTCCAATTGTTAATATAGCATAAATTATTCCTA/3InvdT/
