# Supplementary material for: Direct nucleic acid analysis of mosquitoes for high fidelity species identification and detection of Wolbachia using a cellphone
Source: PLoS Negl Trop Dis. 2018 Aug 30;12(8):e0006671. doi: 10.1371/journal.pntd.0006671 (PMC6116922; doi:10.1371/journal.pntd.0006671)
Supplement: S1 Fig — LAMP uses 2 inner (FIP and BIP) and 2 outer (F3 and B3) primers specific to 6 blocks of target sequences designated as B3, B2, B1, F1c, F2c and F3c. F2 sequence in FIP (F1c-F2) initiates amplification by Bst DNA polymerase (Stage I). F1c sequence in FIP self-primes subsequent amplification. Similarly, BIP (B1c-B2) initiates DNA synthesis by binding to B2c. F3 and B3 primer-initiated DNA synthesis displaces preceding inner primer-initiated strands, which serve as templates for primer-initiated strand displacement DNA synthesis (Stage II). 3′-ends of the resulting single-stranded, dumbbell-shaped amplicons (Stage III) are extended by Bst polymerase to form hairpins (Stage IV). Inner primers hybridize to the single-stranded loops and initiate another round of strand displacement synthesis that opens the original hairpin to form a concatemerized amplicon containing a self-priming 3′-end hairpin (Stage V). The ensuing continuous amplification (initiated both by new inner primers and by self-priming) generates increasingly complex, double-stranded concatameric amplicons containing self-priming hairpins and single-stranded loops to which the OSD probe hybridizes. “c”: denotes complementary target sequences. F and Q on the OSD denote fluorophore and quencher, respectively. OSD probe is denoted in terms of numbered domains, each of which represents a short fragment (usually <12 nt) of DNA sequence in an otherwise continuous oligonucleotide strand. Single stranded toeholds are numbered in red. Complementarity between numbered OSD domains is denoted by a single prime symbol. (PDF) [file pntd.0006671.s002.pdf]

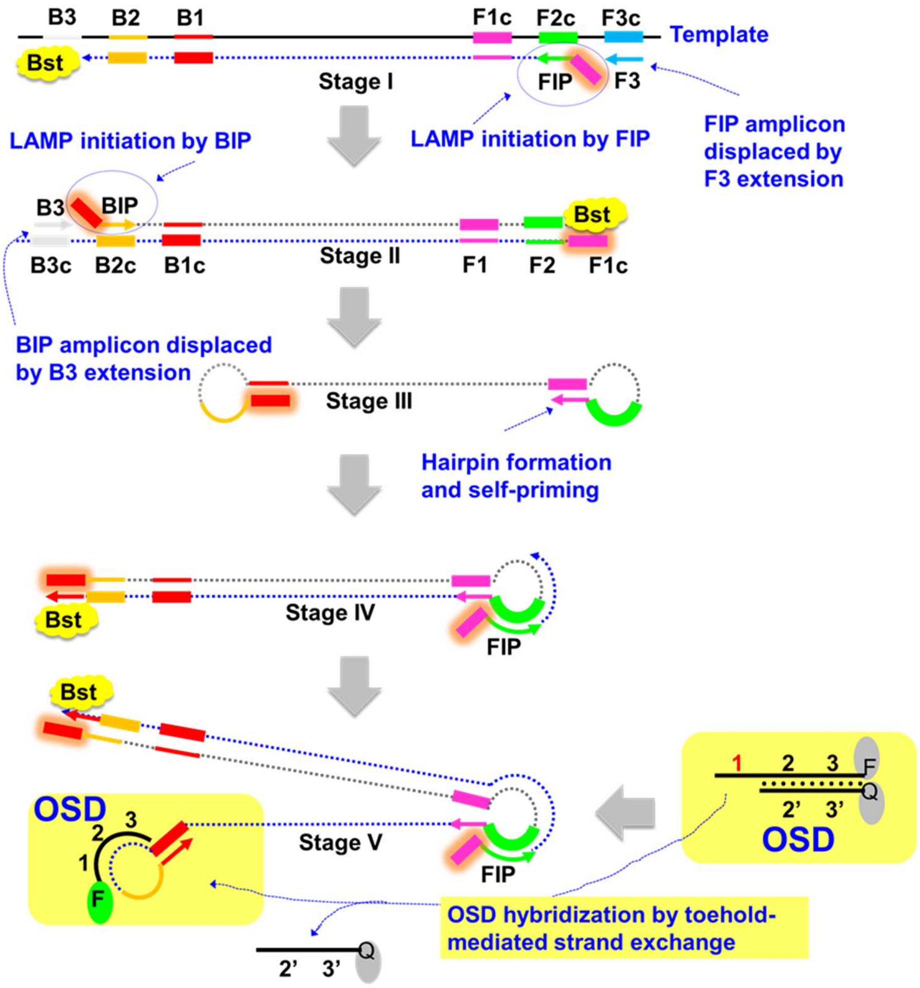

S1 Fig. LAMP-OSD schematic. LAMP uses 2 inner (FIP and BIP) and 2 outer (F3 and B3) primers specific to 6 blocks of target sequences designated as B3, B2, B1, F1c, F2c and F3c. F2 sequence in FIP (F1c-F2) initiates amplification by Bst DNA polymerase (Stage I). F1c sequence in FIP self-primes subsequent amplification. Similarly, BIP (B1c-B2) initiates DNA synthesis by binding to B2c. F3 and B3 primer-initiated DNA synthesis displaces preceding inner primer-initiated strands, which serve as templates for primer-initiated strand displacement DNA synthesis (Stage II). 3′-ends of the resulting single-stranded, dumbbell-shaped amplicons (Stage III) are extended by Bst polymerase to form hairpins (Stage IV). Inner primers hybridize to the single-stranded loops and initiate another round of strand displacement synthesis that opens the original hairpin to form a concatemerized amplicon containing a self-priming 3′-end hairpin (Stage V). The ensuing continuous amplification (initiated both by new inner primers and by self-priming) generates increasingly complex, double-stranded concatameric amplicons containing self-priming hairpins and single-stranded loops to which the OSD probe hybridizes. “c”: denotes complementary target sequences. F and Q on the OSD denote fluorophore and quencher, respectively. OSD probe is denoted in terms of numbered domains, each of which represents a short fragment (usually <12 nt) of DNA sequence in an otherwise continuous oligonucleotide strand. Single stranded toeholds are numbered in red. Complementarity between numbered OSD domains is denoted by a single prime symbol.
