# Supplementary material for: Direct nucleic acid analysis of mosquitoes for high fidelity species identification and detection of Wolbachia using a cellphone
Source: PLoS Negl Trop Dis. 2018 Aug 30;12(8):e0006671. doi: 10.1371/journal.pntd.0006671 (PMC6116922; doi:10.1371/journal.pntd.0006671)
Supplement: S2 Fig — Alignment using wAlbB as the reference strain (A). “Current wsp OSD” refers to the wsp OSD probe used in the present study that binds to the loop sequence between the F1 and F2 target regions. It would not distinguish the closely related wAlbB and wPip strains. The “wAlbB vs wPip OSD”, which would bind to the loop region between the B1 and B2 regions of our current wsp LAMP assay would allow discrimination of wAlbB and wPip strains due to specificity of interaction with the three highlighted polymorphic positions. The wsp sequences of the remaining Wolbachia strains are significantly different from wAlbB wsp sequence and are not detected by the wAlbB/wPip-specific wsp LAMP-OSD assay (B). Pooled synthetic DNA representing wAlbA, wAus, wMors, and wAna as well as standalone wAlbB wsp target sequences were analyzed by wAlbB/wPip-specific wsp LAMP-OSD assays. Smartphone image was acquired at endpoint following 90 min of amplification. (PDF) [file pntd.0006671.s003.pdf]

A

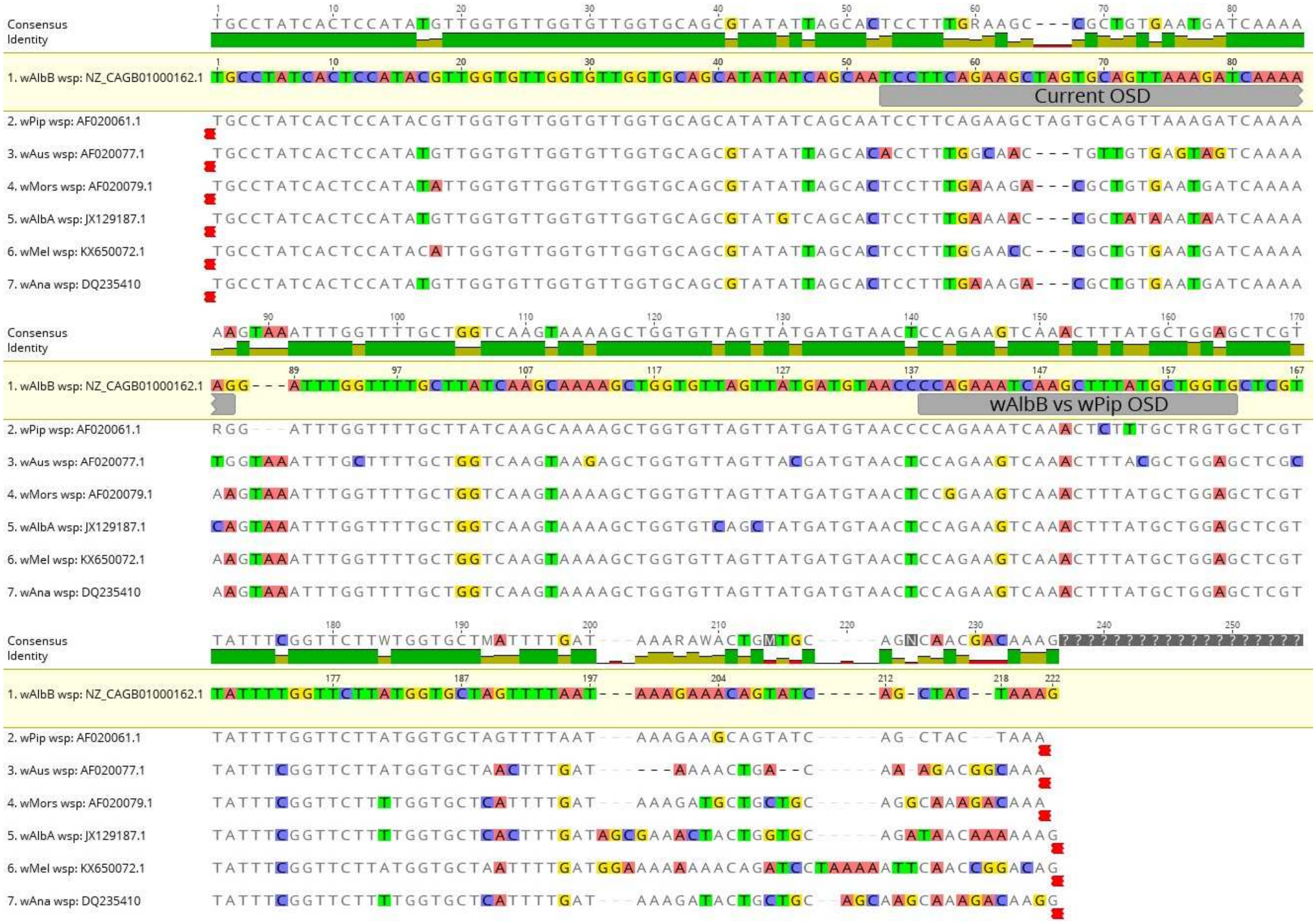

B

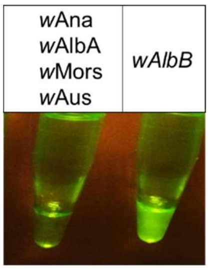

S2 Fig. Comparison of *wsp* sequences from different *Wolbachia* strains. Alignment using *w*AlbB as the reference strain (**A**). "Current *wsp* OSD" refers to the *wsp* OSD probe used in the present study that binds to the loop sequence between the F1 and F2 target regions. It would not distinguish the closely related *w*AlbB and *w*Pip strains. The "*w*AlbB vs *w*Pip OSD", which would bind to the loop region between the B1 and B2 regions of our current *wsp* LAMP assay would allow discrimination of *w*AlbB and *w*Pip strains due to specificity of interaction with the three highlighted polymorphic positions. The *wsp* sequences of the remaining *Wolbachia* strains are significantly different from *w*AlbB *wsp* sequence and are not detected by the *w*AlbB/*w*Pip-specific *wsp* LAMP-OSD assay (**B**). Pooled synthetic DNA representing *w*AlbA, *w*Aus, *w*Mors, and *w*Ana as well as standalone wAlbB *wsp* target sequences were analyzed by *w*AlbB/*w*Pip-specific *wsp* LAMP-OSD assays. Smartphone image was acquired at endpoint following 90 min of amplification.
