# Supplementary material for: Direct nucleic acid analysis of mosquitoes for high fidelity species identification and detection of Wolbachia using a cellphone
Source: PLoS Negl Trop Dis. 2018 Aug 30;12(8):e0006671. doi: 10.1371/journal.pntd.0006671 (PMC6116922; doi:10.1371/journal.pntd.0006671)
Supplement: S3 Fig — Wolbachia wMel-infected Drosophila melanogaster (A), Wolbachia uninfected Culex tarsalis (B), Wolbachia wPip infected Culex quinquefasciatus (Houston) (C) and Culex quinquefasciatus (Salvador) (D) were analyzed using the wsp LAMP-OSD assay. 2 μL of crudely crushed individual insect samples (crushed individual fruit flies were resuspended in 20 μL water) were subjected to LAMP amplification for 90 min. OSD fluorescence was imaged at endpoint using a cellphone. (PDF) [file pntd.0006671.s004.pdf]

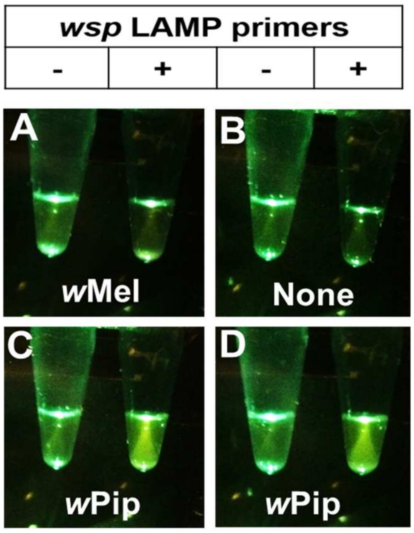

S3 Fig. Detection of *Wolbachia w*Pip using *wsp* LAMP-OSD assay. *Wolbachia w*Mel-infected *Drosophila melanogaster* **(A)**, *Wolbachia* uninfected *Culex tarsalis* **(B)**, *Wolbachia w*Pip infected *Culex quinquefasciatus* (Houston) **(C)** and *Culex quinquefasciatus* (Salvador) **(D)** were analyzed using the *wsp* LAMP-OSD assay. 2 µL of crudely crushed individual insect samples (crushed individual fruit flies were resuspended in 20 µL water) were subjected to LAMP amplification for 90 min. OSD fluorescence was imaged at endpoint using a cellphone.
