# Supplementary material for: Direct nucleic acid analysis of mosquitoes for high fidelity species identification and detection of Wolbachia using a cellphone
Source: PLoS Negl Trop Dis. 2018 Aug 30;12(8):e0006671. doi: 10.1371/journal.pntd.0006671 (PMC6116922; doi:10.1371/journal.pntd.0006671)
Supplement: S4 Fig — Indicated copies of recombinant plasmids bearing coi or wsp target sequences were amplified by the coi-specific (A) or wsp-specific (B) LAMP-OSD assays, respectively. 8% volume of all LAMP-OSD reactions was composed of crudely ‘in-syringe’ prepared non-specific mosquito sample. OSD fluorescence was imaged at endpoint using a cellphone. (PDF) [file pntd.0006671.s005.pdf]

A ***coi* LAMP-OSD**

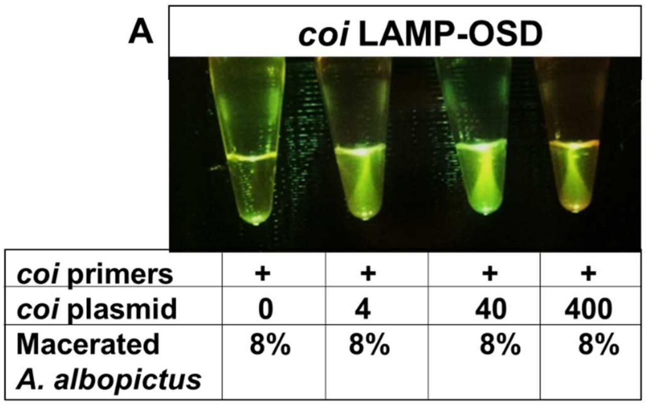

***coi* primers**	**+**	**+**	**+**	**+**
***coi* plasmid**	**0**	**4**	**40**	**400**
**Macerated *A. albopictus***	**8%**	**8%**	**8%**	**8%**

B ***wsp* LAMP-OSD**

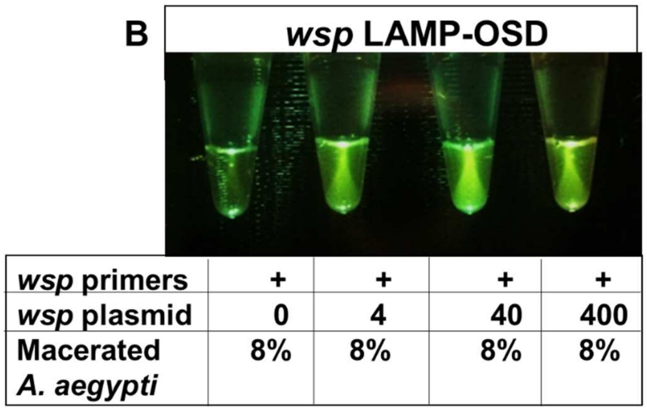

***wsp* primers**	**+**	**+**	**+**	**+**
***wsp* plasmid**	**0**	**4**	**40**	**400**
**Macerated *A. aegypti***	**8%**	**8%**	**8%**	**8%**

S4 Fig. Effect of crude mosquito sample on the detection limit of visually-read LAMP-OSD. Indicated copies of recombinant plasmids bearing *coi* or *wsp* target sequences were amplified by the *coi*-specific **(A)** or *wsp*-specific **(B)** LAMP-OSD assays, respectively. 8% volume of all LAMP-OSD reactions was composed of crudely ‘in-syringe’ prepared non-specific mosquito sample. OSD fluorescence was imaged at endpoint using a cellphone.
