# Supplementary material for: Direct nucleic acid analysis of mosquitoes for high fidelity species identification and detection of Wolbachia using a cellphone
Source: PLoS Negl Trop Dis. 2018 Aug 30;12(8):e0006671. doi: 10.1371/journal.pntd.0006671 (PMC6116922; doi:10.1371/journal.pntd.0006671)
Supplement: S5 Fig — Mosquitoes stored for one week at the indicated temperatures were prepared by ‘in-tube’ crude processing. 2 μL of a 1:10 dilution of each mosquito sample was analyzed by coi LAMP-OSD (A) and by wsp LAMP-OSD (B) assays. OSD fluorescence was imaged at endpoint using a smartphone. (PDF) [file pntd.0006671.s006.pdf]

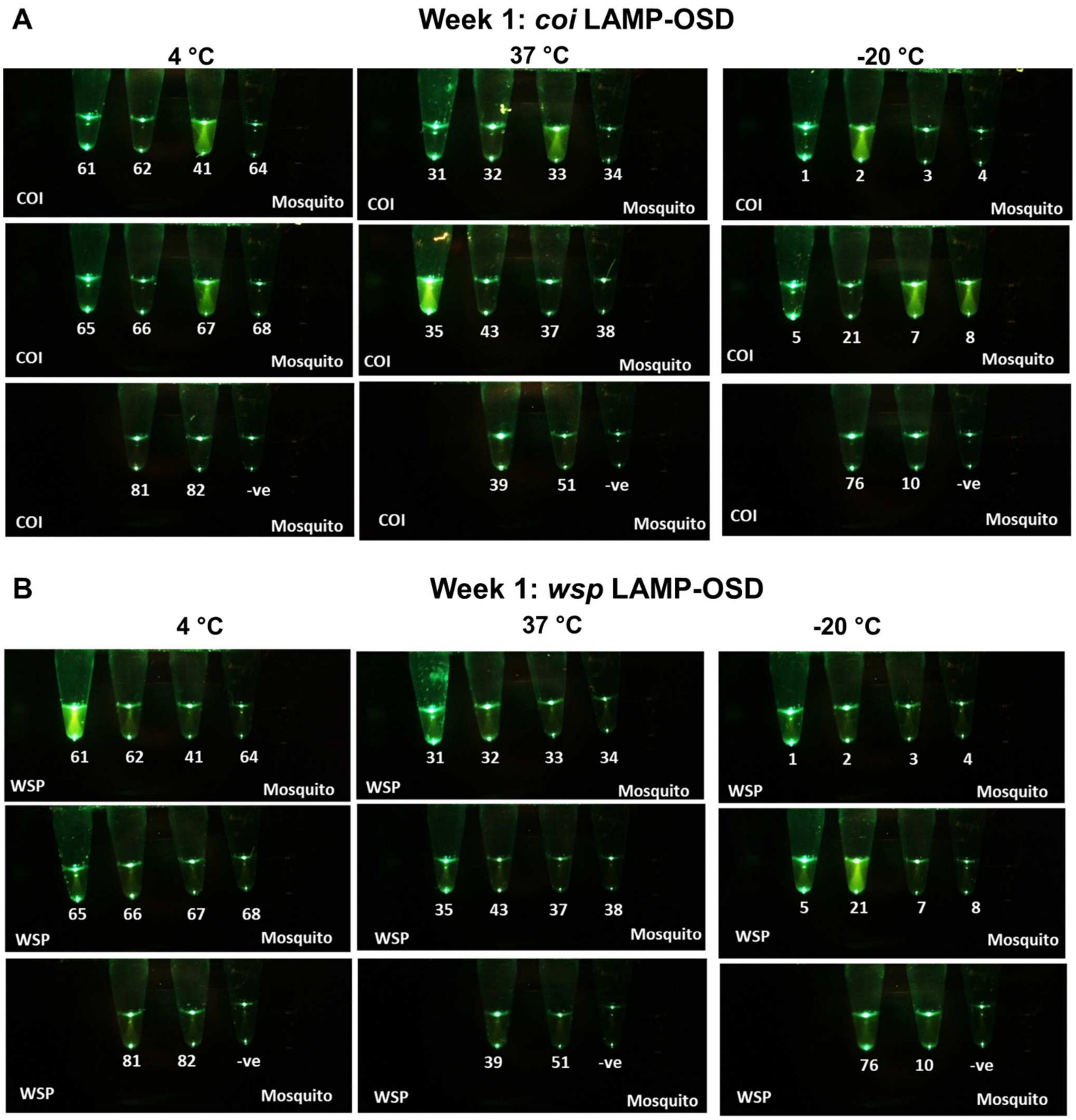

S5 Fig. Blinded LAMP-OSD analysis of field-caught mosquitoes. Mosquitoes stored for one week at the indicated temperatures were prepared by 'in-tube' crude processing. 2 µL of a 1:10 dilution of each mosquito sample was analyzed by *coi* LAMP-OSD (A) and by *wsp* LAMP-OSD (B) assays. OSD fluorescence was imaged at endpoint using a smartphone.
